# Serial-omics of P53−/−, Brca1−/− Mouse Breast Tumor and Normal Mammary Gland

**DOI:** 10.1038/s41598-017-15132-y

**Published:** 2017-11-06

**Authors:** Susanne B. Breitkopf, Mateus De Oliveira Taveira, Min Yuan, Gerburg M. Wulf, John M. Asara

**Affiliations:** 1 Beth Israel Deaconess Medical Center, Division of Signal Transduction, Boston, MA USA; 2 Harvard Medical School, Department of Medicine, Boston, MA USA; 3 Division of Hematology and Oncology, Beth Israel Deaconess Medical Center, Boston, Massachusetts USA

## Abstract

This study demonstrates a liquid-liquid extraction for the sequential tandem mass spectrometry (LC-MS/MS) analysis of non-polar lipids, polar metabolites, proteins and phosphorylation sites from a single piece of tissue. Extraction of 10 mg BRCA−/−, p53−/− breast tumor tissue or normal mammary gland tissue with methyl-tert-butyl ether (MTBE) results in three phases: an upper non-polar phase containing 1,382 lipids, a lower polar phase with 805 metabolites and a precipitated protein pellet with 4,792 proteins with 1,072 phosphorylation sites. Comparative analysis revealed an activated AKT-mTOR pathway in tumors. Tumors also showed a reduction of phosphorylation sites involved in transcription and RNA splicing and decreased abundance of enzymes in lipid synthesis. Analysis of polar metabolites revealed a reduction in glycolysis, pentose phosphate pathway, polyamines and nucleotides, but an increase in TCA and urea cycle intermediates. Analysis of lipids revealed a shift from high triglycerides in mammary gland to high phospholipid levels in tumors. The data were integrated into a model showing breast tumors exhibit features on the proteomic, lipidomic and metabolomic level that are distinct from normal breast tissue. Our integrative technique lends itself to samples such as tumor biopsies, dried blood spots and fluids including urine and CSF to develop biomarkers of disease.

## Introduction

Proteomics, metabolomics and lipidomics provide increasingly robust information due to advances in high resolution mass spectrometry and computational methods^1,2^. Efforts have been made to implement these technologies into human clinical care to expand diagnoses, treatment and disease prevention. One of the challenges is to combine the different levels of -omics comprehensively and consistently working with small samples, such as fine needle aspirates, and to avoid distortion due to over-processing of the sample.

It is important for a complete view on diseases such as cancer to investigate changes on multiple levels of biological activity as each one gives a unique but partial profile. Hence, integration of information on proteomic, lipidomic and metabolomic levels promises to provide a novel, multi-dimensional view of cellular activity. Our lab has demonstrated the integration of phosphoproteomics using stable isotope labeling, non-polar lipidomics and polar metabolomics including labeled metabolic flux analysis in BCR-ABL H929 myeloma cells to generate a biological model of the drivers of cancer cell growth^3^. Polar metabolomics provides a broad overview over pathways in central carbon metabolism such as glycolysis, pentose phosphate pathway and TCA cycle as well as other metabolites such as amino acids, nucleotides, degradation pathways, etc.^4,5^. Recent work using mass spectrometry-based targeted metabolomics profiling has provided a number of insights into how and why these metabolic processes are rewired in cancer^6–8^. The top layer from a liquid extraction with MTBE contains nonpolar lipids required as structural and functional components of membranes, energy storage within lipid droplets, and intracellular and extracellular signaling molecules^9^. Lipidomics profiling typically requires high-resolution MS to obtain the high mass accuracy required to accurately identify head groups and fatty acid chains, including mass shifts due to differences in saturation of fatty acid chains^10–13^. Proteomics and phosphoproteomics have been performed using tandem mass spectrometry for over two decades to identify and quantify both proteins and their post-translational modification sites for biomarker discovery and signaling function^14–17^. Phosphoproteomics illustrates the differences of disease tissue versus normal tissue in regards to kinase signaling that drives tumor growth^17,18^. Researchers have demonstrated one vial extraction methods for performing multiple –omics from a single sample^19–22^. For example, Comen *et al*. showed proteomics, metabolomics and lipidomics data from a MTBE extraction from cell lines and Salem *et al*. showed metabolite, protein and lipid extraction data from plants using MTBE^21^.

In this study, we show a comprehensive serial study from MTBE extractions from mouse breast tumor tissue and mammary gland tissue samples where we identified and quantified more than 4,792 proteins, 1,072 unique phospho-Ser/Thr/Tyr containing peptides, 805 polar metabolites and 1,382 non-polar lipids from 10 mg of starting material using tandem mass spectrometry (LC-MS/MS). We utilized a combination of high resolution and triple quadrupole mass spectrometry for both untargeted and targeted analyses. This method offers a chance to evaluate metabolites, lipids, phosphopeptides and proteins from a single liquid-liquid extraction without technical variation in handling. It provides the possibility to illustrate the biological pathways in a tumor that drive its growth. This strategy could form the basic concept for using mass spectrometry based –omics technologies to identify potential new therapeutic targets and helping to identify the most effective treatment. In the age of personalized medicine, gathering a collection of biomarkers specific to an individual patient’s tumor is important to formulate future treatment. Up until now, the large quantities of tissue for multiple extractions rendered this approach unfeasible for clinical use. Here we present a method that allows the detection of the non-genetic molecular biology of tumor samples on the proteomic, lipidomic and metabolomic level in a single step.

## Results

We analyzed breast tumor and normal mammary tissue from the same mouse in triplicate in order to develop a strategy that can be applied to tumor biopsied samples or dried blood spots using a comprehensive one-step methyl tert-butyl ether (MTBE) based liquid-liquid extraction protocol for serial-omics^19,20^. It creates a MTBE, methanol/water and solid phase separation where the non-polar lipids are soluble in the upper phase, the aqueous polar metabolites are soluble in the middle phase and proteins, long DNA/RNA and cell debris are found as a precipitated insoluble pellet at the bottom of the tube. This method is fast, simple and straightforward with a high reproducibility and can be scaled according to the sample size or volume. The extraction efficiency is comparable to commonly used methods (80% methanol for metabolites, chloroform/methanol for lipids, acetone or TCA precipitation for proteins)^19,20^. We started with 10 mg of mouse breast tumor tissue and 10 mg of mouse mammary gland tissue from the same mouse (K14-Cre BRCA1f/fp53f/f female)^23^ and propagated in syngeneic littermate as described^24–26^.

**Figure 1:**
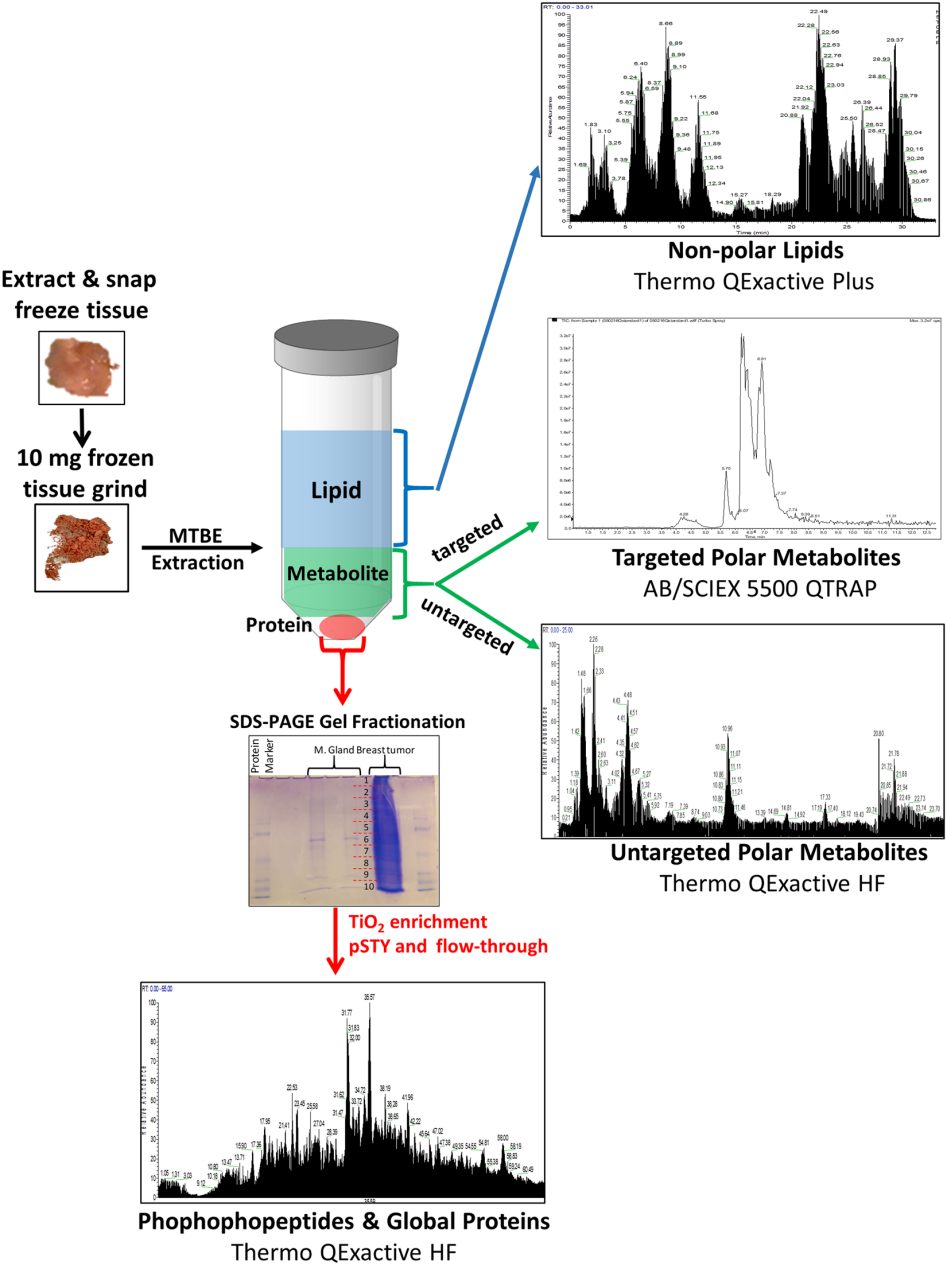
Workflow of the Serial-omics experiment. 10 mg of breast tumor tissue and 10 mg of normal mammary gland were harvested from the mouse and snap frozen separately in liquid nitrogen. The frozen tissue was ground to a powder over dry ice. The powder was solubilized in PBS and extracted with methyltert-butyl ether (MTBE), methanol and water. The upper non-polar liquid phase was collected, dried out and analyzed for untargeted lipidomics via DDA with a Thermo QExactive Plus high resolution Orbitrap mass spectrometer. The lower liquid phase was collected, dried out and analyzed with polarity switching for polar metabolomics via both targeted metabolomics (AB/SCIEX 5500 QTRAP hybrid triple quadrupole mass spectrometer) and untargeted metabolomics (high resolution Thermo QExactive HF Orbitrap). The precipitated protein pellet was re-suspended in sample buffer and separated via a SDS-PAGE gel, fractionated, digested with trypsin and enriched for phosphopeptides via TiO_2_. The digestion mixture and TiO_2_ enriched phosphopeptides were analyzed by DDA in pos mode with a Thermo QExactive HF.

The tumor was allowed to grow to approximately 1 cm^3^ (approximately 2–3 weeks), when the mouse was euthanized and the tumor was immediately snap frozen in liquid nitrogen. The extraction was performed according to the MTBE extraction protocol published by *Matyash et al*.^20^ and Breitkopf *et al*.^27^ with frozen breast tumor or mammary tissue ground in 200 µL PBS, 1.5 mL methanol, 5 mL MTBE and 1.25 mL HPLC grade water. The two liquid phases were collected separately and dried while a solid protein precipitate remained at the bottom on the extraction tube (Fig. 1).

### Upper phase

The dried pellet from the non-polar upper liquid phase was re-suspended in 30 µL 50/50 isopropanol/methanol (w/w) and injected on a reverse phase C_18_ HPLC column to a high resolution QExactive Plus Orbitrap mass spectrometer in a data dependent analysis (DDA) with positive/negative polarity switching^27^. The data was identified and quantified using LipidSearch with the integrated database and Elements software (NIST/HMDB database), annotated with LipidMaps and further processed with MetaboAnalyst software^28^. LipidSearch software first identifies lipid based on an internal library of masses and fragment ions from fatty acid chains, and head groups. Lastly, the software can perform alignment of samples cohorts in order to accurately quantify the MS1 peak profiles^29^.

### Middle phase

The dried samples from the middle polar liquid phase were re-suspended in 20 µL water and one part was injected onto an amide-HILIC HPLC column coupled to a hybrid triple quadrupole QTRAP 5500 mass spectrometer via selected reaction monitoring (SRM) with ~300 targets with positive/negative switching, and integrated using MultiQuant software^4^. A second aliquot of the middle phase was analyzed by a reverse phase C_18_ column coupled to a high resolution QExactive HF Orbitrap mass spectrometer using data dependent analysis (DDA) with positive/negative polarity switching for untargeted profiling. Metabolites were identified by Elements with integrated spectral library databases. Further statistical and pathway analysis from both targeted and untargeted data was performed with MetaboAnalyst.

### Lower solid phase

The pellet containing proteins were re-solubilized and loaded and run via SDS-PAGE gel, coomassie blue stained and cut into 10 equal fractions and digested with trypsin/LysC (Fig. 1). After peptide extraction, the phosphopeptides were enriched with TiO_2_ pipet tips. The phosphopeptides and the flow through (non-phosphorylated peptides) of the TiO_2_ tips from each gel fraction were analyzed separately by a reverse phase C_18_ column coupled to a high resolution QExactive HF Orbitrap mass spectrometer in positive mode via DDA. The peptides and proteins were identified by Mascot and Scaffold Q+S software using the UniProt Mouse protein database.

#### Lipidomics analysis of p53−/−, Brca1−/− breast tumor

**Figure 2:**
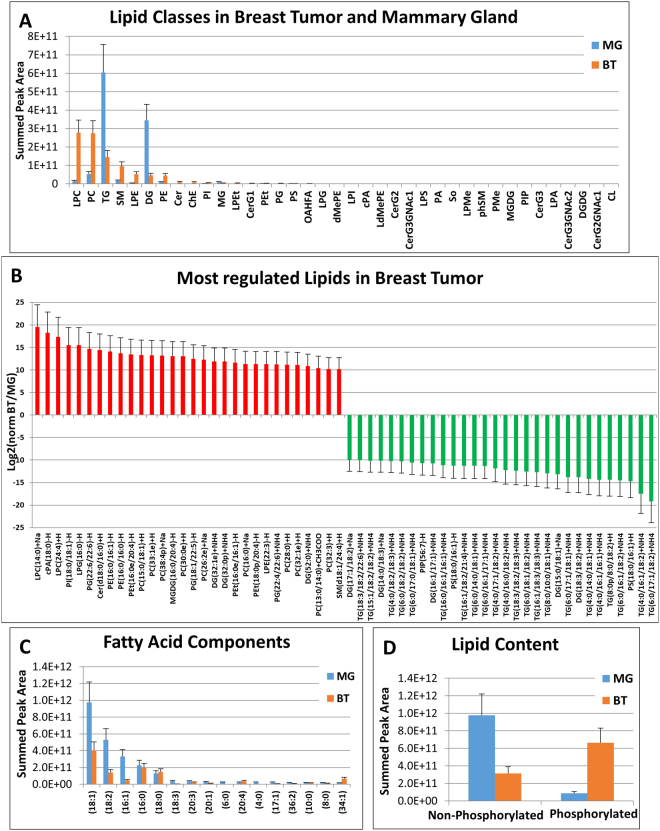
Results from the non-polar lipid fraction. (**A**) The overall lipid class profile across all identified lipids from mammary gland and breast tumor tissue. (**B**) A bar plot of the most regulated lipid ions from the breast tumor vs. mammary gland experiment. (**C**) The fatty acid chain profile for all lipids in the breast tumor and mammary gland tissue. (**D**) The bar plot of the intensity of non-phospho vs. phospholipids suggests a higher abundance of non-phosphorylated lipids in mammary gland and a larger quantity of phospholipids in breast tumor.

The lipidomics platform can identify and quantify lipids from 18 main lipid classes and 66 sub classes including fatty acids, glycerophospholipids, triglycerides, cardiolipins, etc. in a global untargeted, data dependent manner. For MS1 peak and MS2 fragment ion peaks integration, identification and quantification we used LipidSearch Using this approach, we identified and quantified 1,306 unique intact lipid ions. We also performed a spectral library matching search against the NIST (www.nist.gov/srd) MS/MS database using Elements software. We identified a total of 1,383 lipids using both software strategies. While the majority of lipids were shared between the breast tumor and mammary gland tissues, there were some that were uniquely found in cancer tissue. There were significant lipid class differences between tissue types. We discovered that the glycerophospholipid classes such as LPC, PC, LPE, and PE were elevated in breast tumor samples compared to mammary gland (Fig. 2A,D). Comparatively, mammary gland samples had very high levels of triglycerides (TG) and diacylglycerols (Fig. 2B). These findings correlate with the composition previously described^30^, where TG are the main composition (95%) in normal mammary gland tissue but makes up only 25% of all lipids in the mammary carcinomas^31^. Additionally, unsaturated fatty acids such as palmitoleate (C16:1), oleate (C18:1) and linoleate (C18:2) are elevated in mammary gland. These results are typical of tissues with a high fat component^30^. The saturated fatty acids stearate (C18:0) and palmitate (C16:0) showed little difference between breast tumors and mammary glands (Fig. 2C). In agreement with Rees *et al*., we also observed the main difference being a higher phospholipid level in breast tumor cells and higher triglyceride levels in the mammary gland tissues (Fig. 2D)^32^. These data suggest that proliferating tumor cells generate cellular lipids to support the generation of membranes needed for mitosis and cell growth and to produce phospholipids required for signaling, while the mammary gland’s main function is storage for fat. Supporting Online Dataset I contains all lipid identifications from LipidSearch and Elements software^27^.

#### Metabolomics analysis of P53−/−, Brca1−/− breast tumor

We employed two metabolomic platforms capable of identifying hundreds of unique endogenous polar metabolites including SRM based QTRAP technology and a global non-targeted platform from MS and MS/MS spectral library matching using a high resolution QExactive Orbitrap mass spectrometry. The results from each metabolomics analysis were combined and produced 804 unique metabolites including potential isomers (254 from the targeted analysis) and covering all major central carbon metabolism pathways as well as many others including amino acids, nucleotides, vitamins, redox, ROS, etc. (Supporting Online Dataset II). The majority of unique metabolites were again detected in the breast tumor, similar to the lipidomics datasets although the vast majority of detected metabolites were shared between tissue types.

**Figure 3:**
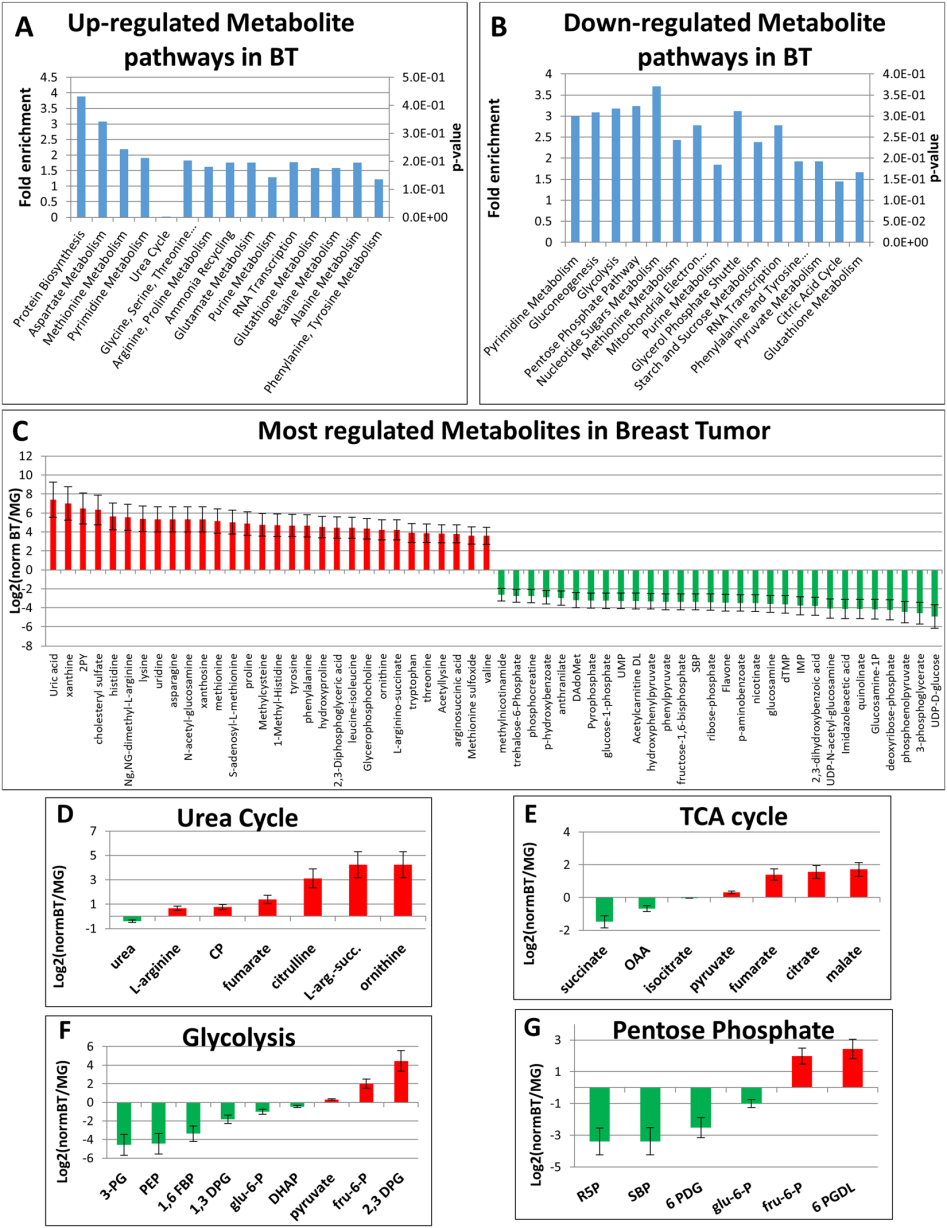
Results from the polar metabolite fraction. (**A**) The biological pathway profile with fold enrichment across all up-regulated metabolites including p-values generated with MetaboAnalyst software. (**B**) The pathway profile with fold enrichment across all down-regulated metabolites including p-values generated by MetaboAnalyst. (**C**) A bar plot of the most regulated metabolites from the breast tumor vs. mammary gland experiments. (**D**) A bar plot for detected metabolites of the urea cycle, elevated in breast tumors. (**E**) A bar plot for detected metabolites of the TCA cycle. (**F**) A bar plot for detected metabolites in the glycolysis pathway. (**G**) A bar plot for detected metabolites of the pentose phosphate pathway.

**Figure 4:**
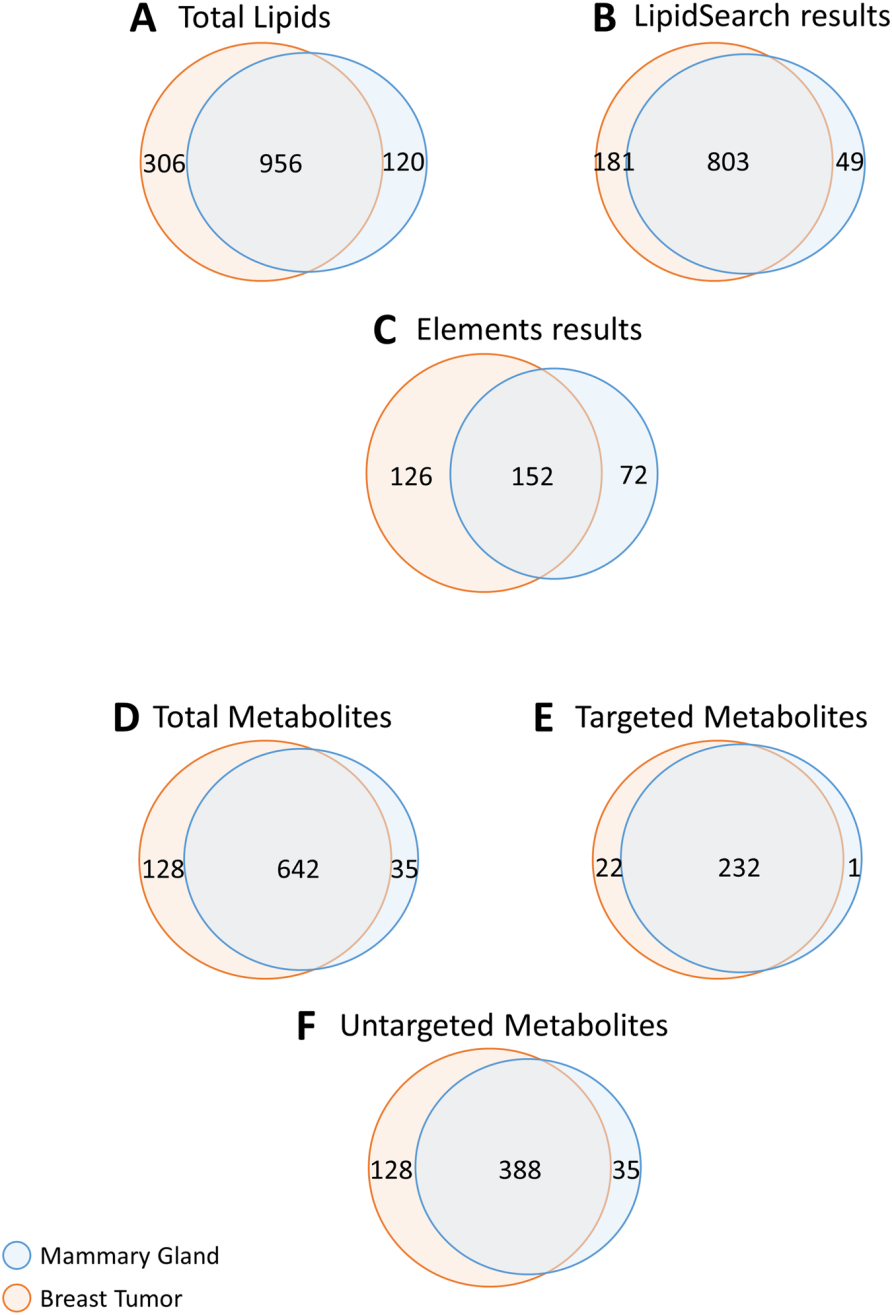
Venn diagrams for the distribution of unique small molecules. (**A**) The venn diagram shows the total amount of unique lipids (1,382) identified from the serial-omics of mouse breast tumor and mouse mammary gland by LC-MS/MS. (**B**) The lipid number distribution identified with LipidSearch (1,033) and (**C**) Elements (350) from mouse breast tumor and mammary gland. (**D**) The venn diagram shows the total amount of unique metabolites (805) identified from the serial-omics from mouse breast tumor and mammary gland by LC-MS/MS. (**E**) The targeted metabolite number distribution (254) and (**F**) untargeted metabolite number (551) distribution identified with Elements from the mouse breast tumor and mouse mammary gland.

We identified distinct profiles for both mammary gland and breast tumor including a high level of amino acids such as histidine, lysine, methionine, proline and tyrosine in breast tumor cells compared to mammary tissue (Fig. 3A,C). From the targeted LC-MS/MS data, the most highly up-regulated metabolites in the breast tumor tissue were uric acid, xanthine and several members involved in protein biosynthesis such as basic amino acids. The most down-regulated metabolites were UDP-glucose and several intermediates of glycolysis. The untargeted LC-MS/MS metabolomics data showed amino acids including methionine and related metabolites as well as taurine as the most highly elevated in breast tumor. The most down-regulated metabolites in tumor were organic acids and tri-amino acids involved in protein biosynthesis and purine metabolism. Additionally, metabolites of the urea cycle such as arginine, citrulline carbamoyl phosphate and ornithine were highly concentrated in breast tumor (Fig. 3A,D) however, polyamines generated from ornithine and the urea cycle showed a higher level in mammary glands. For the central carbon metabolism pathways, the TCA cycle and glycolysis demonstrated mixed regulation within the major pathways. For the TCA cycle, mostly higher levels were observed in breast tumor with up-regulated malate, citrate, isocitrate and fumarate though succinate and oxaloacetate were slightly down-regulated in tumors (Fig. 3A,E). The amino acids generated from the TCA cycle such as arginine, glutamine, glutamic acid and asparagine were all up-regulated in the breast tumor, consistent with increased TCA cycle activity (Fig. 3E). In contrast, many of the glycolytic intermediates including glucose-6-phosphate, 1,6-fructose-bisphosphate, dihydroxyacetone phosphate, 3-phosphoglycerate and phosphoenolpyruvate were down-regulated in breast tumor (Fig. 3B,C and F). Interestingly, pyruvate and lactate were increased in the breast tumor tissue. That may indicate that glycolytic flux is greater than consumption and efflux of pyruvate and lactate, the end products of glycolysis. Similar to the glucose-derived glycolysis pathway, the pentose phosphate pathway (PPP) intermediates from both oxidative and non-oxidative arms were lower in mouse breast tumor (Fig. 3B,C and G). In contrast to amino acids, mononucleotides showed elevated levels in the mammary gland but not in breast tumor. Figure 4A–F shows the distribution of both lipids and metabolites identified with LipidSearch and Elements across the breast tumor and mammary gland tissues. The data shows that the mammary gland contained the majority of unique lipids while the breast tumor contained the majority of unique polar metabolites.

#### Proteomics analysis of p53−/−, Brca1−/− breast tumor

**Figure 5:**
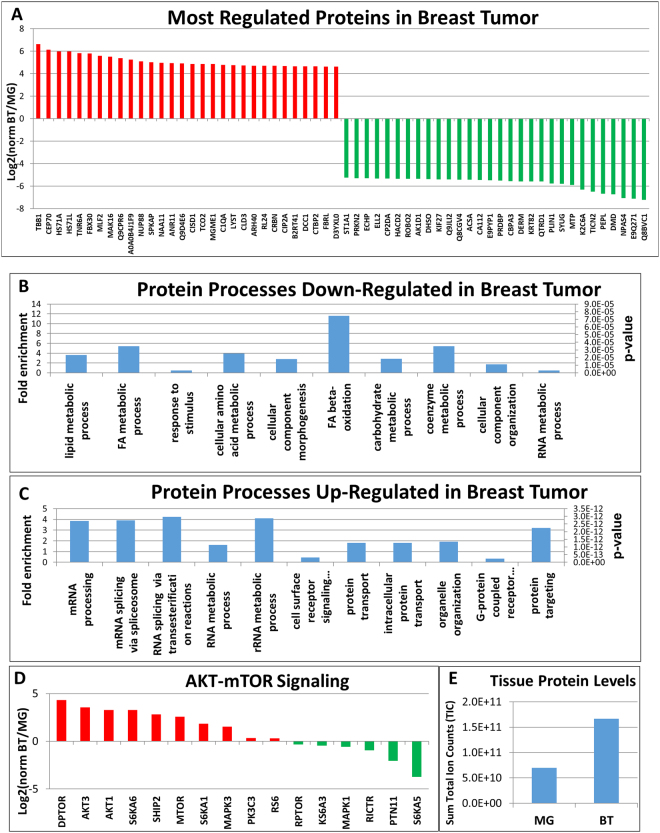
Results from the protein pellet. (**A**) A bar plot of a portion of the most regulated proteins from the breast tumor vs. mammary gland experiment. Down-regulated proteins, such as PLIN1 and ACSA in breast tumor tissue are involved in lipid metabolism. (**B**) The profile for biological processes with fold enrichment across all down-regulated proteins including p-values generated with the Panther Classification System. (**C**) The profile for biological processes with fold enrichment across all up-regulated proteins including p-value generated with Panther Classification System. (**D**) A bar plot with all detected proteins involved in the mTOR pathway. (**E**) A bar blot representing the total protein levels in both breast tumor and mammary gland tissue by summing the MS2 total ion count intensity over all identified proteins (peptides).

The protein precipitate was solubilized for and separated by SDS-PAGE into ten fractions, digested with trypsin, enriched by titanium dioxide (TiO_2_) resin packed tips for phospho-peptide enrichment and both the phosphopeptide enriched samples as well as the flow through of the TiO_2_ enrichment were analyzed by reversed-phase (C_18_) LC-MS/MS using a high resolution QExactive HF Orbitrap mass spectrometer via higher energy dissociation (HCD) with (Top 10, DDA) coupled to a nanoflow HPLC. The peptide sequence and protein identification and label free quantification via spectral counting^33^ was performed via the Mascot search engine and Scaffold Q+S software. We could identify 4,792 unique proteins and 1,071 unique phosphorylation sites from 10 mg of combined breast tissues (Supporting Online Dataset III). It is worth noting that the total protein level in 10 mg of tissue was significantly different between the breast tumor and mammary gland which was also reflected in both the SDS-PAGE gel and by the summed total ion counts (TIC) over all identified proteins by LC-MS/MS (Fig. 5E). An equal mass of breast tumor contained a 7-fold higher protein content than the mammary gland. In breast tumor, we identified 2,728 unique proteins as opposed to only 149 unique proteins in the mammary gland with an overlap of 1,915 proteins between tissue types.

The tumor tissue showed a distinct protein expression profile compared to the mammary gland (Fig. 5A–C). Proteins from the most regulated biological process that were found elevated in mammary gland tissue or down-regulated in breast tumor were involved in lipid and fatty acid metabolism (beta oxidation) according to Panther gene ontology classification. These included proteins such as fatty acid synthase (FASN), carboxylesterase 1 (CES1D), and fatty acid binding protein 4 (FABP4) (Fig. 5B). De novo fatty acid synthesis depends on the production of acetyl coenzyme A (CoA) from glucose through the action of ATP citrate lyase (ACLY)^34^ which was also found elevated in mammary gland. Additional down-regulation can be found for the CoA carboxylase (ACACA), the enzyme catalyzing carboxylation of acetyl-CoA to malonyl-CoA^34^, FASN, which produces palmitate from malonyl-CoA^34^ and fatty acid binding protein 4 (FABP4), which is involved in fatty acid transport. Further, we found key proteins in triglyceride storage and lipolysis PLIN1 and PLIN4 down-regulated on protein level in breast tumor, which confirms previous findings in breast tumors (Fig. 5A,B)^35^. Taken together, these key proteins support reduced de novo lipid synthesis in breast tumor compared to mammary gland as determined by lipidomics. However, it also shows that mammary gland tissue is synthesizing triacylglycerides at high levels and the presence of tumor tissue interferes with normal breast’s ability to produce lipids needed for milk production. We also identified down-regulated tumor proteins which have been associated with poor prognosis in breast cancers including monoacylglycerol lipase (MGL), AHNAK, MYOSIN-1C, guanine nucleotide binding protein GNAI1, serum deprivation response protein SDPR, EH domain containing EHD2 protein, Annexin A1, SPOCK2, Polymerase 1 and transcript release factor Catalase as well as a down-regulation of GAPDH an enzyme supporting the mostly down-regulated glycolysis pathway in the breast tumor, which has also apoptotic properties^36^. The most up-regulated individual proteins in the breast tumor compared to mammary gland are involved in basic cellular building blocks and the cytoskeleton such as tubulin, heat shock, actin, etc. (Fig. 5A). Therefore, we focused on proteins involved in biological pathways with a statistically significant P value according to Panther gene ontology. These included proteins involved in mRNA processing including splicing such as shi-related sequence 4 (SRS4), SRS10, splicing factor 3a subunit 1 (SF3A1), Splicing factor, suppressor of white-apricot homolog (SFSWA) (Fig. 5A), SRSF1 and SRSF4. Their overexpression has been associated with transformation of normal mammary cells to breast cancer cells^37^. More to the point, Y-box proteins (Fig. 5A) activate transcription and mRNA metabolism including transcription, RNA splicing, mRNA stability and translation in low concentrations (Fig. 5C). This leads to the suspicion of alternative splicing in our breast tumor model, which has been described as a crucial function in the survival of cancer cells, and making spliceosome inhibitors effective treatments^38^. In regards to kinase signaling, up-regulation on the protein level in the tumor was detected on both upstream and downstream of the mTOR including AKT1, PI3Kα, GRB2, mTOR, DPTOR, S6K and S6 (Fig. 5D), which is known to be highly mutated in cancer^39^. The consequences of this up-regulated pathway was seen in regulation of transcription factors and splicing factors, inducing the gene expression of the glucose transporter GLUT1/3^40^. Pathways such as mTORC1 have a huge impact on transcription factors such as the up-regulation of transcriptional corepressor TIF1b and the down-regulation of transcriptional activators PurA and PurB^41^ (Fig. 5B). Furthermore the mTORC1 complex inhibits lipolysis^42^ and fatty acid synthesis and changes the TG concentration and fat storage in the tumor^43^, which we already revealed on the lipid level. We could isolate important up-regulation of the breast tumor marker, HSP90 and its interactor Fkbp4^44^, which chaperons mutated and overexpressed oncogenes, causing decreased survival rate of cancer patients (Fig. 5E)^45^. This up-regulation expands to other potential tumor markers including A1AT1, tropomyosin-4, TUBB3, ACTN4, DDX3X, LMNB1, PARP and vimentin.

#### Phosphoproteomics analysis of p53−/−, Brca1−/− breast tumor

**Figure 6:**
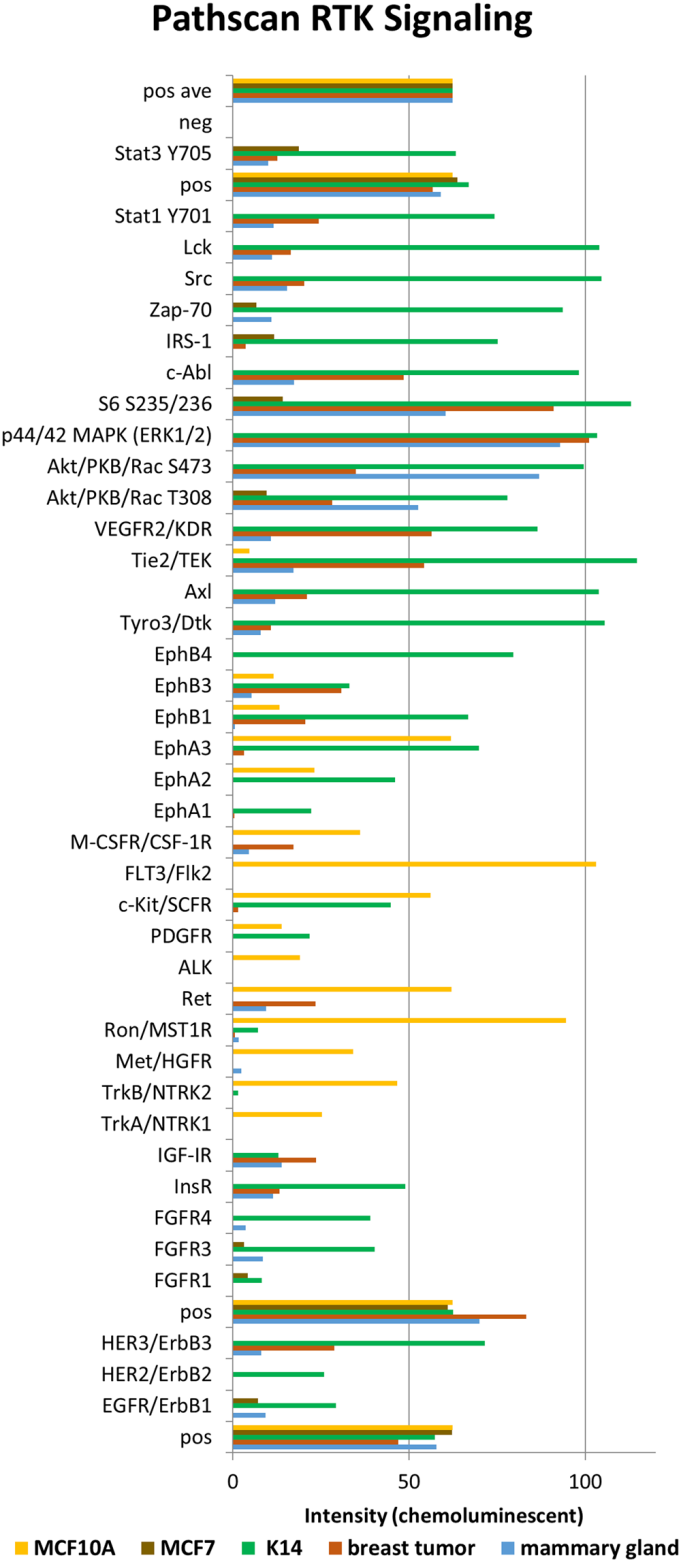
PathScan RTK Array. PathScan RTK Signaling antibody array kit tests the phosphorylated tyrosine and serine/threonine kinase signaling of 39 different kinases from p53−/−, Brca1−/− breast tumor, normal mammary gland, K14 breast cancer cells, MCF7 breast cancer cells and the MCF10A normal breast cell line.

We first performed an activity assay of key signaling proteins including receptor tyrosine kinases (RTKs) and other important signaling molecules using the commercially available PathScan RTK Signaling Array antibody kit that includes 28 receptor tyrosine kinases and 11 important signaling nodes (Fig. 6). The assay was performed on the breast tumor and mammary gland tissue as well as the K14 breast cancer cells, MCF10A normal mammary gland cell line and MCF7 breast cancer cells. The data was then normalized between tissue types based on the control spots on the array. The pan-phosphotyrosine sites on EGFR, which is essential for ductal development in mammary gland^46^ as well as FGFR3, a fibroblast growth factor essential for normal mammary gland development^47^ are both up-regulated in mouse mammary gland tissue. Additionally, the important phospho-AKT T308 signaling site responsible for cell growth and proliferation is up-regulated in mammary gland which is somewhat surprising. The signal for phospho-ERK1/2 on the PathScan array was found to be up-regulated in breast tumor, somewhat expected in tumor development and it is consistent with the total ERK up-regulation in the LC-MS/MS tumor data. This also suggests that the P53−/−, Brca1−/− tumors signal predominantly through ERK and are less dependent upon AKT for kinase signaling. Interestingly, the protein level of AKT was higher in breast tumor according to our LC-MS/MS data. The S6 phosphorylation sites S235/236 were found up-regulated in breast tumor according to PathScan, which also correlate with the increased S6K protein intensity found in the LC-MS/MS data. Highly elevated PathScan signaling molecules in breast tumor compared to normal mammary gland included phospho-RET (pan Tyr), which is known to be up-regulated in estrogen receptor-positive breast cancers^48^ and Tie2 pan Tyr phosphorylation which has been correlated with poor overall survival and high metastasis risk^49^. The complete results of the PathScan analysis from all breast tissues and cell types are shown in Supporting Online Dataset IV.

**Figure 7:**
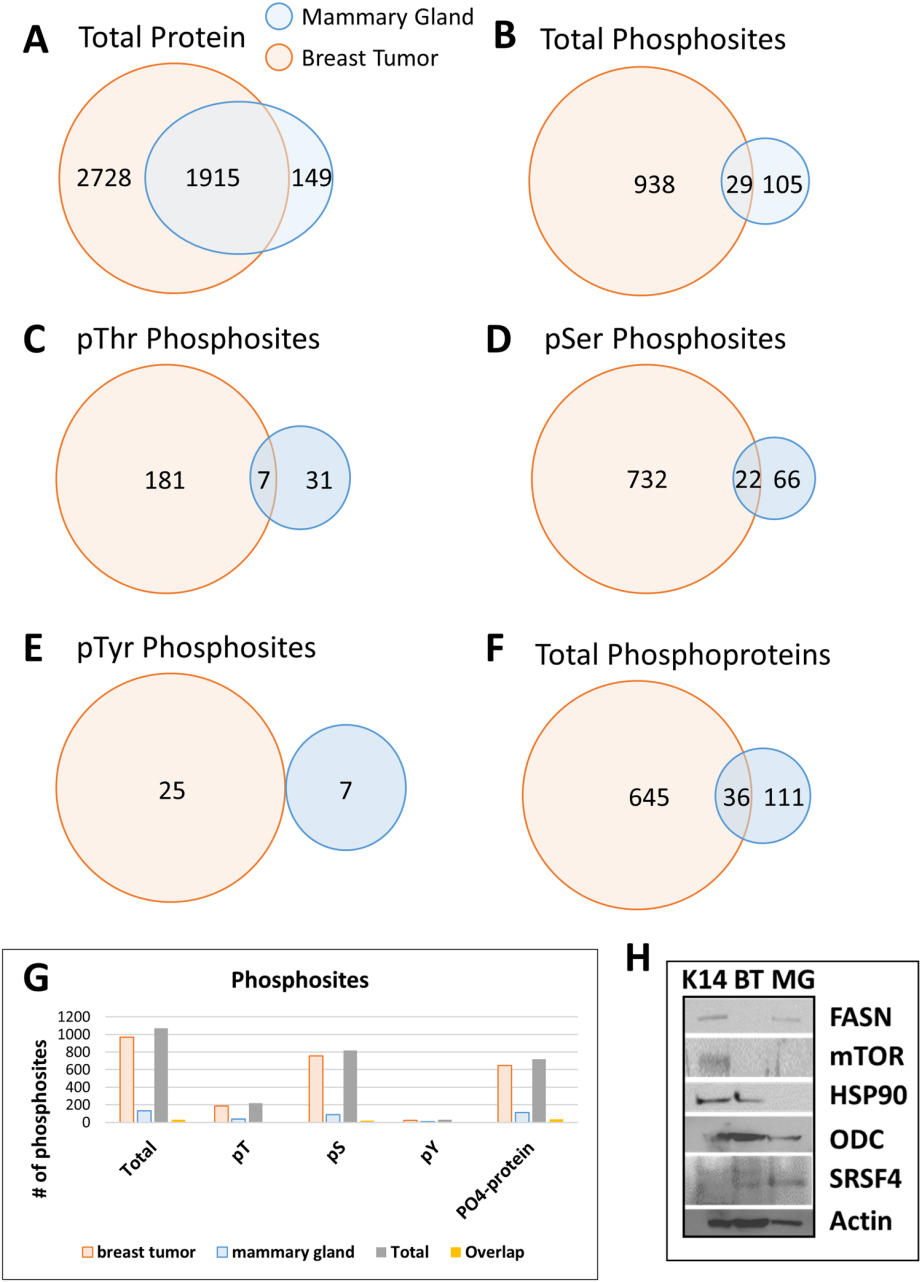
Venn diagrams for the distribution of proteins and peptides. (**A**) The venn diagram shows the total amount of unique phosphosites (1,072) identified from the serialomics of mouse breast tumor and mouse mammary gland by LC-MS/MS. (**B**) The venn diagram shows the total amount of unique phosphoproteins (792) identified from the serialomics of mouse breast tumor and mouse mammary gland by LC-MS/MS. (**C**) The venn diagram shows the total amount of unique phosphothreonine, (**D**) phosphoserine, and (**E**) phosphotyrosine sites identified in mouse breast tumor and mouse mammary gland. (**F**) The venn diagram shows the total amount of unique proteins (4,792) identified from the serial-omics of mouse breast tumor and mouse mammary gland by LC-MS/MS. (**G**) The bar plot represents the amounts of total phosphosites, phospho-threonine, - serine and -tyrosine as well as phosphoproteins in mouse breast tumor and mouse mammary gland. (**H**) Immunoblots of the important signaling proteins FASN, mTOR, HSP90, ODC and SRSF4 with actin loading control.

**Figure 8:**
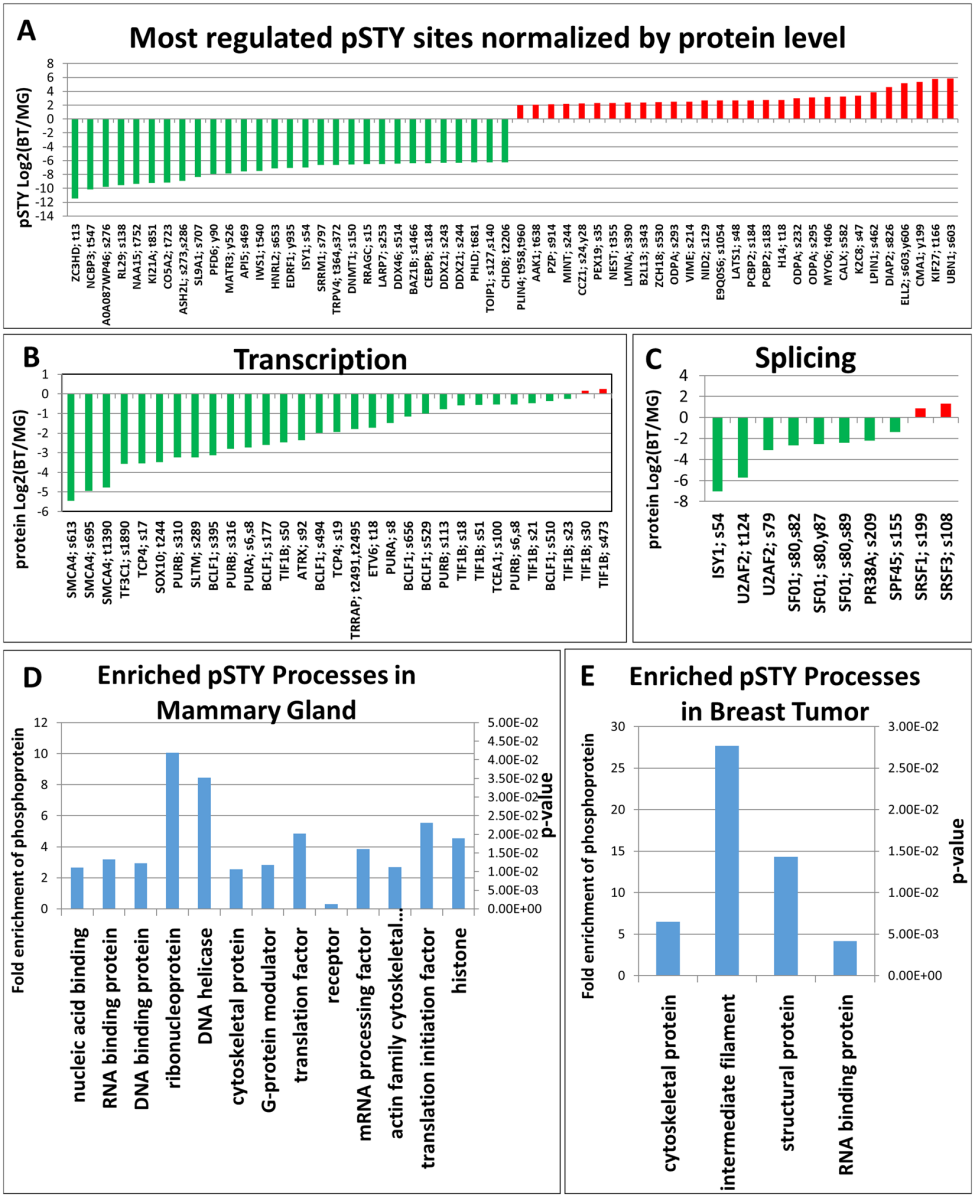
Results from the phosphopeptide enriched protein pellet. (**A**) A bar plot of the most regulated pSTY phoshosites normalized by protein level from the breast tumor vs. mammary gland experiment. (**B**) A bar plot of detected transcription factors in the phosphopeptide enriched fraction. (**C**) A bar plot of proteins involved in RNA splicing from the identified phosphopeptides. (**D**) The profile of protein classes with fold enrichment across all enriched proteins in mammary gland including p-values generated with Panther. (**E**) The profile of protein classes with fold enrichment across all enriched proteins in breast tumor including p-values.

The gel separated and TiO_2_ enriched phosphoproteomics data revealed a vast difference in the amount of phosphorylation sites identified in each tissue sample. From a total of 1,072 phosphosites, the breast tumor contained a total of 938 unique phosphosites with 732 Ser, 181 Thr and 25 Tyr residues. The mammary gland contained only 105 unique phosphosites in total with 66 Ser, 31 Thr and 7 Tyr phosphorylation sites (Fig. 7B–G). The phosphosites could be found on 645 different proteins in BT and 111 unique proteins in MG with an overlap of only 36 (Fig. 7F). It is not surprising that the majority of phosphorylation sites were detected in the breast tumor tissue rather than the mammary gland since cancers are known to show high levels of kinase signaling which drive their growth^50^. Specific to the LC-MS/MS phosphoproteomics dataset (Fig. 8A), the upregulated pSTY data in mouse breast tumor tissue includes S293 on Pdha1 (Pyruvate dehydrogenase E1 component subunit alpha) which regulates the activity of the enzyme and further induces the activity of upregulated sites (S232, S295)^51^. S462 on Lipin1 was up-regulated in breast tumor and is an important regulator of fatty acid metabolism and induced in response to DNA damage and glucose deprivation. Lipin1 regulates gene expression involved in fatty acid oxidation and catalyzes the conversion of phosphatidic acid (PA) to diacylglycerol, a key step in the biosynthesis of triacylglycerol (TG). T638 on AP2 associated kinase 1 (AAK1) showed up-regulation and can regulate fatty acids through LDL-receptor expression regulation and endocytosis^52^. Sites T958/T960 on PLIN4 were up-regulated in tumor, and with PLIN1, is responsible for lipid droplet formation in the biogenesis of lipid droplets^35^. These data help support that normal lipid biosynthesis processes in mammary glands become dysregulated in breast tumors.

Fewer phosphosites were down-regulated in breast tumor tissue or enriched in mammary gland. The large tumor suppressor kinase 1 (LATS1) was down-regulated on both the protein level and on site S48 in breast tumor. Other down-regulated phosphosites were found on proteins that play a regulatory role in transcription and splicing including ubinuclein-1, UBN1 (S603), Poly(RC) binding protein 2, PCBP2 (S183/184), RNA polymerase II elongation factor, ELL2 (S603/Y606) and MINT (S244). Most phosphorylation sites on trascription factors were down-regulated in breast tumor (Fig. 8B), although their protein levels were elevated. The same effect was noted on proteins involved in RNA splicing and their phosphosites (Fig. 8C). Figure 8D shows the enriched protein processes in the mammary gland tisssue while Fig. 8E shows protein processes enriched in the breat tumor exclusively from the regulated phosphoproteomics data. The complete set of identified phosphorylation sites, their regulation and the corresponding proteins can be found in Supporting Online Dataset V. The complete list of enriched Panther biological processes from the proteomic and phosphoproteomic datasets in the breast tumor with P values are shown in Supporting Online Dataset VI.

**Figure 9:**
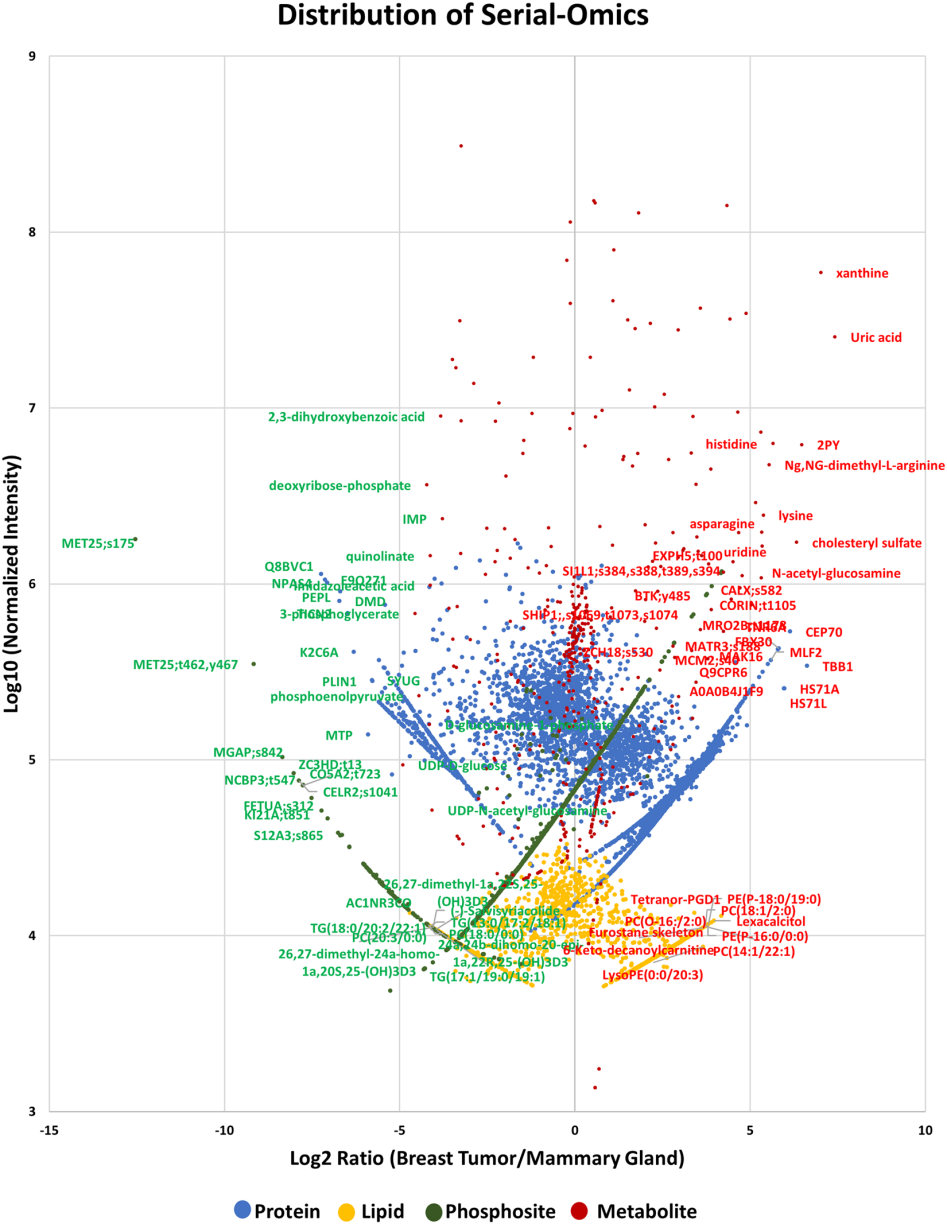
Overlayed scatterplot of metabolomics, lipidomics, proteomics and phosphoproteomics datasets. A scatterplot showing the distribution of the various -omics results from the MTBE extraction of the breast tumor vs. mammary gland experiment. Datasets were normalized for viewing purposes.

**Figure 10:**
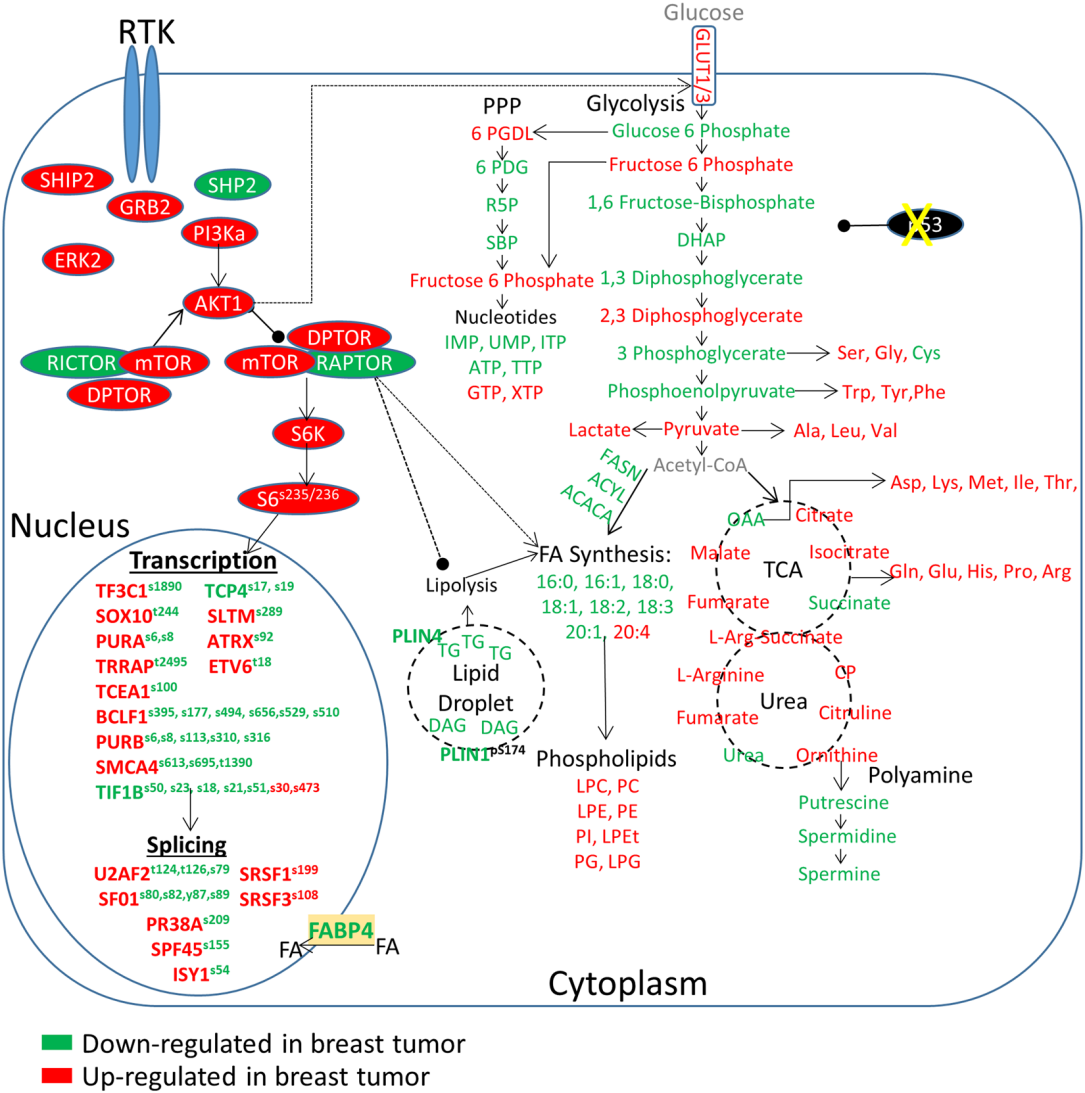
Model of the breast tumor vs mammary gland serial-omics experiment. The MTBE liquid-liquid extraction of the solid protein fraction revealed an active mTOR pathway and regulation on transcription and splicing in the breast tumor. Proteins involved in lipid metabolism were found to be downregulated in breast tumor. The top layer lipid fraction exposed a reduction in lipid biosynthesis and triglyceride levels but an increase in phospholipid levels in breast tumor. The middle layer revealed higher levels of polar metabolites in the urea cycle, TCA cycle and the amino acids in the breast tumor, however, the pentose phosphate pathway and many glycolytic intermediates were reduced in breast tumors.

In order to assemble and integrate the data from five different –omics analyses (1. Untargeted lipidomics, 2. Targeted metabolomics, 3. Untargeted metabolomics, 4. Untargeted proteomics and 5. Untargeted phosphoproteomics) from a single piece of tumor or mammary gland, we annotated a virtual cell based on signal transduction processes, metabolism, lipid biosynthesis, and RNA transcription and protein synthesis (Figures 9-10). The integration was performed based on informatics obtained from each individual analysis and assembled based on knowledge of the current literature. We used multiple pieces of software to bioinformatically assemble the model. Figure 9 shows the overlay as a scatterplot of all omics data and the normalized regulation of each analysis as a single plot. It shows that some –omics data has tighter regulation than others and also shows differences in the dynamic range for each analysis. The metabolomics data shows the widest range of scattered regulation, mostly from the untargeted analysis while the lipidomics data shows the tightest amount of scatter within the dataset. The proteomics and phosphoproteomics datasets showed the most dramatic regulated signals as displayed as bands of data points since many phosphosites and proteins were only detected in either breast tumor or mammary gland but not both. Figure 10 shows the serial-omics data integration which describes the regulation of members of connecting biological pathways including central metabolism, kinase signaling, RNA transcription and splicing, amino acid and nucleotides as well as fatty acid and lipid synthesis in mouse P53−/−, Brca1−/− breast tumor and normal mammary gland tissue.

## Discussion

The biggest advantage of the MTBE extraction lies in one simple and quick extraction, which separates tissue samples for three different -omics from a single sample of small quantity. This makes the possibility for finding biomarkers from needle biopsies less than 10 mg, dried blood spots less than 10 μL, etc. possible. We showed that only 10 mg of tissue was sufficient enough to cover our targeted metabolomics profile list of ~290 important central carbon metabolites of the cell but we further identified 500 additional metabolites by performing the untargeted data dependent metabolomics approach, exposing important changes in breast tumor metabolism compared to mammary gland. Our untargeted lipidomics approach routinely identifies 1,000–1,500 lipid molecules across all major lipid classes. We not only acquire lipid class and fatty acid chain information but the exact composition of the lipid ion including its saturation level. Lastly, the combination of in-gel digestion and TiO_2_ phosphopeptide enrichment from 10 mg per tissue extracted nearly 5,000 proteins and more than 1,000 phosphosites from minimal fractionation. The integration of these multiple –omics data from the same small piece of sample has major advantages since variability across different tissue samples has been eliminated, thus increasing reproducibility. The ability to incorporate data from multiple -omics experiments into a cellular pathway model is extremely powerful for developing a therapeutic strategy for a personalized medicine approach. The first thing that was noted by comparing the serial-omics of 10 mg of breast tumor and mammary gland tissue was the different levels between large and small molecules. The ratio of lipids between breast tumor versus mammary gland was approximately 1:1, the ratio for metabolites is ~2:1 in favor of breast tumor and the most dramatic difference was in the protein level which was ~7:1 in favor of the breast tumor and a ~20 times greater level for phosphopeptides in the breast tumor. However, very different classes of lipids and small molecules were present between the two tissue types as was a same with proteins identified.

We identified activated signaling pathways in the p53−/−, Brca1−/− breast tumor with an especially elevated ERK-MTOR-S6 pathway, which contributes to activation changes on proteins involved in transcription and splicing. Furthermore, we revealed a connection between protein regulation and small molecule metabolites. In the breast tumor, important members of de-novo lipid synthesis were down-regulated on both the lipid and protein level, and the activated mTOR pathway contributed to inhibition of lipolysis. Despite the increased expression of the glucose transporter GLUT1/3, the glycolysis and pentose phosphate pathways were mostly decreased although amino acids, urea and TCA cycle intermediates showed elevation in breast tumor tissue compared to normal mammary gland. The proteomic and RTK data suggest a possible intervention in these tumors with ERK inhibitors.

Taken together, the results from our serial-omics approach exposed the molecular mechanism and signal pathways in the p53−/−, Brca1−/− breast tumor cells. Our model demonstrates the power of acquiring and assembling four types of *–omics* data derived from a single piece of tissue. It shows that an enormous amount of data can be captured from a specimen without wasting any precious tissue. We were capable of detecting known breast tumor suppressor and marker proteins and molecules from a small sample amount equivalent to a needle biopsy. In the future, this approach could be used to help understand the driving mechanism in cancers and help to personalize the treatment of cancer patients from biopsied samples and dried blood spots and potentially from other fluids such as urine, tears, CSF, synovial fluid, etc.

## Methods

### MTBE Extraction

The mouse breast tumor tissue and mouse mammary gland were treated separately for comparison. Each tissue specimen was snap frozen in liquid nitrogen (−196 °C) right after resection and stored in −80 °C until the extraction. Right before extraction the frozen tissue was ground on dry ice with the help of a pestle and mortar. 10 mg of the tissue grind were transferred into a glass vial (Fisher Scientific, 03-340-47 A) and 200 µL of 1X PBS (RT) were added followed by 1.5 mL HPLC grade methanol and vigorous vortexing for 1 min. After the addition of 5 mL of MTBE, anhydr. 99.8% (Sigma Aldrich, 306975-1L) the samples were shaken for 1 hr at RT. We added 1.2 mL of water, vortexed for 1 min and spun for 10 min. The resulting upper (lipid) and lower (metabolite) phases were collected separately in 1.5 mL glass vials and dried out in a SpeedVac. The protein pellet was resuspended in 200 µL 0.5x sample buffer (6X SDS Sample Buffer (0.375 M Tris pH 6.8, 12% SDS, 60% glycerol, 0.6 M DTT, 0.06% bromophenol blue) transferred to a microcentrifuge tube and dried down to 50 µL in a SpeedVac.

### Proteomics/Phosphoproteomics

The protein samples were loaded on a 4–12% gradient gel (Lonza, #58520) and ran until the loading dye reached the bottom of the gel. The gel was stained GelCode Blue Stain (Fisher Scientific, #PI24590) for 30 minutes and each lane with sample was cut into 10 equal pieces. Gel sections were reduced with 55 mM DTT, alkylated with 10 mM iodoacetamide (Sigma-Aldrich), and digested overnight with TPCK modified trypsin (Promega) at pH = 8.3. Peptides were extracted, dried out in a SpeedVac, resuspended in 10 μL of 50% ACN, 6%TFA and rocked on a shaker for 15 min. The TiO2 TopTip (PolyLC, # TT10TIO) were washed with 50% ACN, 6% TFA, spin at 1500 rpm 0.5 min for four times. The samples were loaded on the TiO2 tips and incubated for 30 min followed by wash with 10 µl 50% ACN, 1% TFA (spin at 1500 rpm 0.5 min), repeated two times, eluted with three times 10 µl 40% ACN, 15% NH_4_OH, added 60 µl buffer A (0.1% formic acid/99.9% water) and dried down to 5 µL. The protein sample was analyzed by positive ion mode LC-MS/MS using a high resolution hybrid QExactive HF Orbitrap Mass Spectrometer (Thermo Fisher Scientific) via HCD with data-dependent analysis (DDA) with 1 MS1 scan followed by 8 MS2 scans per cycle (Top 8). Peptides were delivered and separated using an EASY-nLC nanoflow HPLC (Thermo Fisher Scientific) at 300 nL/min using self-packed 15 cm length × 75 μm i.d. C18 fritted microcapillary columns. Solvent gradient conditions were 120 minutes from 3% B buffer to 38% B (B buffer: 100% acetonitrile; A buffer: 0.9% acetonitrile/0.1% formic acid/99.0% water). MS/MS spectra were analyzed using Mascot Version 2.5.1.0 (Matrix Science) by searching the reversed and concatenated mouse protein database (version 201509, http://www.ebi.ac.uk/uniprot/database/download.html) with a parent ion tolerance of 18 ppm and fragment ion tolerance of 0.05 Da. Carbamidomethylation of cysteine (+57.0293 Da) was specified as a fixed modification and oxidation of Methionine (+15.9949), phosphorylation of Serine/Threonine/Tyrosine (+79.97) as variable modifications. Results were imported into Scaffold Q+S 4.6 software (Proteome Software, Inc.) with a peptide threshold of ~85%, protein threshold of 95%, resulting in a peptide false discovery rate (FDR) of ~1%. Known contaminants such as keratins, caseins, trypsin and BSA were removed from the analysis. Further statistical analysis was performed using Panther (http://www.pantherdb.org/).

### Lipidomics

The lipid samples were re-suspended in 30 µL of 1:1 LC/MS grade isopropanol:methanol prior to LC-MS/MS analysis, 5 µL were injected. A Cadenza 150 mm × 2 mm 3 µm C18 column (Imtakt) heated to 40 °C at 260 µL/min was used with a 1100 quaternary pump HPLC with room temperature autosampler (Agilent). Lipids were eluted over a 20 min. gradient from 32% B buffer (90% IPA/10% ACN/10 mM ammonium formate/0.1% formic acid) to 97% B. A buffer consisted of 59.9% ACN/40% water/10 mM ammonium formate/0.1% formic acid. Lipids were analyzed using a high resolution hybrid QExactive Plus Orbitrap mass spectrometer (Thermo Fisher Scientific) in DDA mode (Top 8) using positive/negative ion polarity switching. DDA data were acquired from m/z 225-1450 in MS1 mode and the resolution was set to 70,000 for MS1 and 35,000 for MS2. MS1 and MS2 target values were set to 5e5 and 1e6, respectively. Lipidomics data were analyzed using LipidSearch 4.1.9 software (Thermo Fisher Scientific) and Elements for Metabolomics (Proteome Software) NIST database incorporated.

### Metabolomics

Half of the metabolite samples were re-suspended in 20 μL LC/MS grade water, 5 μL were injected over a 15 min gradient using a hybrid 5500 QTRAP triple quadrupole mass spectrometer (AB/SCIEX) coupled to a Prominence UFLC HPLC system (Shimadzu) via SRM of a total of 287 SRM transitions using positive/negative polarity switching corresponding to 258 unique endogenous water soluble metabolites. The dwell time was 3 ms per SRM resulting in ∼10–14 data points acquired per detected metabolite. Samples were separated using a Amide XBridge HPLC hydrophilic interaction liquid chromatographic (HILIC) column (3.5 μm; 4.6 mm inner diameter (i.d.) × 100 mm length; Waters) at 300 μL/min. Gradients were run starting from 85% buffer B (HPLC grade acetonitrile) to 40% B from 0–5 min; 40% B to 0% B from 5–16 min; 0% B was held from 16–24 min; 0% B to 85% B from 24–25 min; 85% B was held for 7 min to re-equilibrate the column. Buffer A was comprised of 20 mM ammonium hydroxide/20 mM ammonium acetate (pH = 9.0) in 95:5 water/acetonitrile. Peak areas from the total ion current for each metabolite SRM transition were integrated using MultiQuant version 2.1 software (AB/SCIEX) via the MQ4 peak integration algorithm using a minimum of 8 data points with a 20 sec retention time window.

The other half of the metabolite samples were re-suspended in 30 μL LC/MS grade water, 5 μL were analyzed by positive negative switching mode using a high resolution QExactive HF hybrid quadrupole-Orbitrap mass spectrometer (Thermo Fisher Scientific) via a Top 8 DDA. Metabolites were delivered and separated using an EASY-nLC nanoflow HPLC (Thermo Fisher Scientific) at 225 nL/min using self-packed 15 cm length × 75 μm i.d. C18 fritted microcapillary columns. Solvent gradient conditions were 25 minutes from 3% B buffer to 38% B (B buffer: 100% acetonitrile; A buffer: 0.1% formic acid/99.9% water). The data were analyzed using Elements for Metabolomics (Proteome Software) with the NIST database incorporated (http://chemdata.nist.gov/mass-spc/msms-search/) followed by statistical analysis with Metaboanalyst 3.0 (http://www.metaboanalyst.ca/).

### Normalization of data sets

For normalization each of the three data sets first the average value of all ratios, breast tumor versus mammary gland was calculated followed by multiplying each data point of the mammary gland data set with the calculated average value. Additional normalization was needed for the values of the phosphorylation sites. The ratio of breast tumor versus mammary gland of each phosphosite was divided by the ratio of breast tumor versus mammary gland of the corresponding protein.

### Syngeneic Tumor Implants

All animal experiments were conducted in accordance with Institutional Animal Care and Use Committee-approved protocols at Beth Israel Deaconess Medical Center. Tumors generated in K14-Cre BRCA1f/fp53f/f mice were syngeneically transplanted into the mammary pad of K14-Cre− mice to generate cohorts of mice. Tumors were extracted when they reached 20 mm in diameter.

### Cell culture

The human mammary epithelium cell line MCF7 (Ralph Scully lab, BIDMC) and mouse breast tumor cell line K14 were obtained in Dulbecco’s Modified Eagle’s medium (Corning; DMEM with L-Glutamine, 4.5 g/L Glucose) supplemented with 10% FCS, 100 units/mL penicillin and 100 units/mL streptomycin. MCF10A (Ralph Scully lab, BIDMC) were maintained in MEGM bullet kit (Lonza CC-3150) with 100 ng/ml cholera toxin, 100 units/mL penicillin and 100 units/mL streptomycin.

### PathScan RTK signaling array

The PathScan RTK signaling array kit containing 39 fixed antibodies in duplicates against phosphorylated forms of common key signaling proteins by the sandwich ELISA format was used per manufacturer’s direction (Cell Signaling Technologies). Images were analyzed with ImageJ (http://rsbweb.nih.gov/ij/) by loading the image as a gray scale. Each kinase array dot was manually selected and an average intensity for each kinase was calculated. Normalization within one stimulation experiment was done by subtracting the intensity of the negative control dot from each value. For comparison of different stimulation conditions, sets were normalized so that the positive controls had equal intensities.

### Western Blots

Cells were lysed in lysis buffer (0.5% (v/v) NP-40, 1% (v/v) Triton X-100, 150 mM NaCl, 50 mM Tris·Cl, pH 7.4, 1 mM EDTA, 1 mM EGTA, protease inhibitors), and protein concentration was calculated using Bradford assay. Equal amounts were loaded onto a Gradient Gel 4–20% (Lonza). Western blot analyses were conducted after separation by SDS-PAGE (Gradient Tris-Glycin gel 4–20%, Lonza) and transferred to a nitrocellulose membrane. Antibodies against HSP90 (rabbit polyclonal CS 4874S), mTor (rabbit polyclonal CS 2972), Fatty Acid Synthase (rabbit polyclonal CS 3180), beta-Actin (rabbit polyclonal CS 4970), ODC (rabbit polyclonal, Abcam ab126590), SFRS4 (rabbit polyclonal, Abcam ab73893) PLIN1 (rabbit polyclonal). All antibodies were used per manufacturer’s instructions. Antibody binding was detected using enhanced chemiluminescence (PerkinElmer).

## Electronic supplementary material

Dataset I_Lipidomics

Dataset II_Metabolomics

Dataset III_Proteomics

Dataset IV_PathScan

Dataset V_Phosphoproteomics

Dataset VI_Panther Analysis
